# Exposure to whole-body vibration in open-cast mines in the Barents region

**DOI:** 10.3402/ijch.v75.29373

**Published:** 2016-02-09

**Authors:** Lage Burström, Ville Hyvärinen, Magnar Johnsen, Hans Pettersson

**Affiliations:** 1 Department of Public Health and Clinical Medicine, Occupational and Environmental Medicine Umeå University, Umeå, Sweden; 2 Arcum, Umeå University, Umeå, Sweden; 3 Finnish Institute of Occupational Health, Oulu, Finland; 4 Department of Occupational and Environmental Medicine, University Hospital of North Norway, Tromsø, Norway

**Keywords:** whole-body vibration, mining, drivers, ISO2631-1, measurements

## Abstract

**Objectives:**

We aimed to measure and evaluate whole-body vibration (WBV) exposure among drivers of mining vehicles in the Barents region.

**Study design:**

In the period from November 2012 to August 2014, this cross-sectional study was carried out at 3 mines in Finland, Norway and Sweden as part of the MineHealth project.

**Methods:**

Measurements of WBV were conducted on the surface of the driver's seat during normal work in accordance with international standards. Personal data on daily exposure times were collected by a questionnaire.

**Results:**

Measurements were conducted on 95 different mining vehicles both as root mean square (RMS) value and vibration dose value (VDV) representing different manufacturers, models and capacities. Of the 453 miners who answered the questionnaire, 232 indicated that they were exposed to WBV during their working day. The results show that the mean daily exposure time varies between 1.9 and 6.7 h for different vehicles. The calculated mean A(8) could be found in an interval between 0.2 and 1.0 m/s^2^ and the corresponding 8-h VDV fell between 7 and 17 m/s^1.75^.

**Conclusions:**

Exposure to WBV among operators of mining vehicles may be a serious health and safety problem in the mines studied. The employers ought, therefore, take active steps to reduce exposure in accordance with the European vibration directive. Moreover, since some groups of drivers are exposed to vibration that is close to or exceeds the exposure limit values, the employer should take immediate action to reduce exposure below these values.

Occupational exposure to whole-body vibration (WBV) originating from the operation of vehicles has long been acknowledged to be a significant risk for low back pain for vehicle drivers (1). Operators of vehicles used in open-cast mining are exposed to significant WBV for relatively long periods (2–7). Studies have also indicated that the prevalence of self-reported musculoskeletal pain is higher among exposed groups of miners compared with none-exposed groups (8,9).

In the mining industry, the workers’ tasks have become increasingly mechanized, with more time spent operating machinery and driving vehicles (10), leading to an increase in the proportion of workers exposed to WBV. In the Barents region, there is currently a rapidly growing mining industry where existing mines are being expanded and new mines are being opened. However, the environmental conditions in the Barents region are characterized by considerable fluctuations in temperature with cold and snowy winters and bright summers. These variations affect the road and surface conditions in open-cast mines, which are known to be critical for the exposure to WBV (7). Knowledge of exposure to WBV is, however, limited for the mining industry in circumpolar regions, which emphasizes the need for measures to reduce the burden of workers’ exposures.

The international standard ISO 2631 (11) has been developed for guidance on how WBV should be measured and reported. Moreover, the European vibration directive (12) stipulates minimum requirements for the health and safety of workers exposed to WBV. The European directive specifies 2 daily vibration exposure levels, exposure action value (EAV) and exposure limit value (ELV), in terms of weighted root mean square (RMS) acceleration and vibration dose value (VDV) over an 8-h period. The prescribed limits of 8-h RMS and VDV are for EAV 0.50 and 9.1 m/s^1.75^ and for ELV 1.15 and 21.0 m/s^1.75^, respectively.

In addition, the directive requires employers to reduce worker vibration exposure levels wherever this is practically possible. Where an operator is likely to be exposed to vibration, an assessment of the likely daily vibration exposure is required. If the exposure level is above the EAV, a range of actions must be undertaken in order to reduce exposure and lower the risks. If the ELV is exceeded, immediate action must be taken to reduce vibration exposure below the ELV, and procedures implemented to prevent it being exceeded again.

To assist employers in the evaluation and assessment of risks arising from WBV, a special European standard (13) has been published. This standard provides guidelines for the measurement and evaluation of WBV at the workplace. The employers should perform an assessment of the risk, including estimation of worker daily vibration exposure levels, so as to determine whether the EAV or ELV are likely to be exceeded during normal work with vibrating equipment. It is, therefore, of considerable importance that data on WBV levels and daily exposure are generated to encompass a wide range of equipment and operations.

This work aims to provide data for open-cast mines in the Barents region by measuring and evaluating WBV exposure among operators of mining vehicles in Finnish, Norwegian and Swedish open-cast mines.

## Material and methods

In the period from November 2012 to August 2014, this cross-sectional study was carried out at 3 mines in Finland, Norway and Sweden as part of the MineHealth project (14). The project, funded by the EU′s “Kolarctic European Neighbourhood and Partnership Instrument Cross Border Cooperation (Kolarctic ENPI CBC),” seeks to improve the sustainability of miners’ well-being, health and work ability in the Barents region. The Finnish nickel–copper–platinum mine is located approximately 150 km north of the City of Rovaniemi in Finnish Lapland. The Norwegian nepheline syenite mine is located approximately 40 km north of the City of Alta in Finnmark County, Norway. The Swedish copper mine is located close to the City of Gällivare in Swedish Lapland. The group of mine workers included in the study covers all the different occupations at the mines, with the main groups being drivers of vehicles, mechanics, electricians and supervisors.

In order to conduct comparable measurements in all countries, a common study protocol was constructed (15) and training sessions were conducted for international staff. For taking the measurements, commercially available devices by Larson Davis Human Vibration Monitor HVM/IHVM 100 were used. The instruments provide frequency-weighted measurements of acceleration consistent with ISO 2631-1. WBV was measured on the driver's supporting seat surface during normal work by placing a standardized rubber seat pad containing accelerometers for the 3 orthogonal axes in accordance with ISO 2631-1. A calibrator checked the measuring equipment before and after the measurements.

Accelerometer data were collected while the different vehicles were involved in normal mining production and operated by experienced drivers. The measurement durations ranged from 4 to 198 min (mean 60 min). The exposure duration to WBV has been estimated from self-reported exposures by using a questionnaire that was developed within the MineHealth project and translated into each country's native language (16). The questionnaire also included questions related to health complaints, leisure activities and use of working clothes. The questionnaire was completed by 453 workers (participation rate 67%) who had given their consent for participation.

The assessment of the level of exposure to vibration was based on the calculation of daily exposure A(8) expressed as equivalent continuous acceleration over an 8-h period, calculated as the highest RMS value or the highest VDV of the frequency-weighted accelerations and determined on 3 orthogonal axes (X, Y, Z) in accordance with ISO 2631-1 (11). In the calculation, the values in the X- and Y-directions were multiplied by a factor of 1.4. The following formulae were used:

$$\begin{array}{l} {\text{A}}_{\text{X}}\text{(}8\text{)}=1.4\cdot \text{RM}{\text{S}}_{\text{X}}\cdot \sqrt{\frac{T_{e}}{8}}\\ {\text{A}}_{\text{Y}}\text{(}8\text{)}=1.4\cdot \text{RM}{\text{S}}_{\text{Y}}\cdot \sqrt{\frac{T_{e}}{8}}\\ {\text{A}}_{\text{Z}}\text{(}8\text{)}=1.0\cdot \text{RM}{\text{S}}_{\text{Z}}\cdot \sqrt{\frac{T_{e}}{8}} \end{array}$$

where A(8) for each direction is the 8-h equivalent acceleration, *T*_e_ is the actual exposure time in hours and RMS is the equivalent acceleration during the period *T* hours. The A(8) is then defined as the highest of A_X_(8), A_Y_(8) and A_Z_(8). The calculations of the VDVs over an 8-h period were conducted in the same manner.

All statistical calculations were performed with the statistical program SPSS version 22 (IBM Corp, 2013) and the study was approved by the regional committees for medical research in each of the countries (Finland 2012-73; Norway 2013-1026; Sweden 2012-365-31M).

## Results

The results of the measurements of WBV in the 3 countries among different mining vehicles are summarized in Table I. The table shows the measured vibration both as RMS values and calculated VDVs for the actual mean measurement period in the 3 directions X, Y and Z according to ISO 2631-1.

**Table I:** The mean measured RMS acceleration in the 3 directions X, Y and Z according to ISO 2631-1

Group of vehicle	Number of vehicles	Measuring time (min)	RMS X (m/s^2^)	RMS Y (m/s^2^)	RMS Z (m/s^2^)	VDV X (m/s^1.75^)	VDV Y (m/s^1.75^)	VDV Z (m/s^1.75^)
Haul trucks	37	58	0.26	0.29	0.43	3.2	3.5	5.3
		(39)	(0.06)	(0.09)	(0.13)	(1.0)	(1.4)	(2.0)
		[5–144]	[0.17–0.43]	[0.16–0.51]	[0.18–0.75]	[2.0–5.6]	[1.7–7.1]	[2.8–10.3]
Drilling rigs	7	25	0.14	0.08	0.22	1.6	1.1	4.6
		(10)	(0.14)	(0.06)	(0.16)	(1.3)	(0.6)	(4.6)
		[10–35]	[0.03–0.44]	[0.04–0.20]	[0.05–0.51]	[0.7–4.4]	[0.4–1.9]	[1.4–13.1]
Wheel loaders	15	79	0.62	0.62	0.49	7.9	7.7	7.7
		(46)	(0.19)	(0.19)	(0.18)	(2.8)	(2.8)	(4.5)
		[16–128]	[0.46–0.86]	[0.34–1.06]	[0.28–0.95]	[4.1–14.5]	[4.4–15.0]	[2.9–15.5]
Excavators	18	59	0.37	0.25	0.39	4.7	3.6	6.3
		(37)	(0.12)	(0.06)	(0.17)	(1.9)	(1.2)	(3.8)
		[12–117]	[0.18–0.52]	[0.14–0.34]	[0.16–0.69]	[2.2–7.6]	[1.8–5.5]	[1.8–14.8]
Dozers(wheel, track)	6	72	0.73	0.82	0.70	7.4	7.9	8.7
		(45)	(0.25)	(0.25)	(0.30)	(2.4)	(2.1)	(3.2)
		[35–150]	[0.54–1.22]	[0.51–1.17]	[0.28–1.04]	[4.8–11.6]	[6.2–11.6]	[3.5–12.5]
Graders	4	140	0.26	0.28	0.38	3.5	3.9	4.9
		(72)	(0.08)	(0.15)	(0.06)	(0.9)	(1.3)	(1.3)
		[50–198]	[0.17–0.37]	[0.18–0.50]	[0.33–0.46]	[2.4–4.5]	[2.6–5.6]	[4.1–6.9]
Transport cars	8	19	0.24	0.30	0.71	1.8	2.3	11.4
		(15)	(0.07)	(0.13)	(0.21)	(0.4)	(1.0)	(3.1)
		[5–49]	[0.18–0.39]	[0.16–0.45]	[0.44–1.14]	[1.4–2.5]	[1.0–3.9]	[7.0–15.9]

Number of measurements and mean measuring time carried out for different vehicle groups are given. Calculated VDV corresponding to the mean measuring time for each group of vehicles in the 3 vibration directions is provided. The standard deviation (SD) is given within parenthesis and minimum as well as maximum values within brackets.

Altogether, 95 vibration measurements were taken, with a mean measurement time of 60 min (SD 44.6; range 4–198 min). The drill rig is the equipment that causes the lowest WBV exposure, while wheel loaders, dozers and transport cars provide the highest exposure level expressed as RMS values. For estimated VDVs, the exposure in the vertical Z-direction (up–down) in general causes the highest magnitudes. However, within each group of vehicles, there is a great variety in terms of manufacturer, model and capacity, and the wide standard deviations as well as the range of values also indicate a large variation in measured vibration magnitudes.

**Fig. 1:**
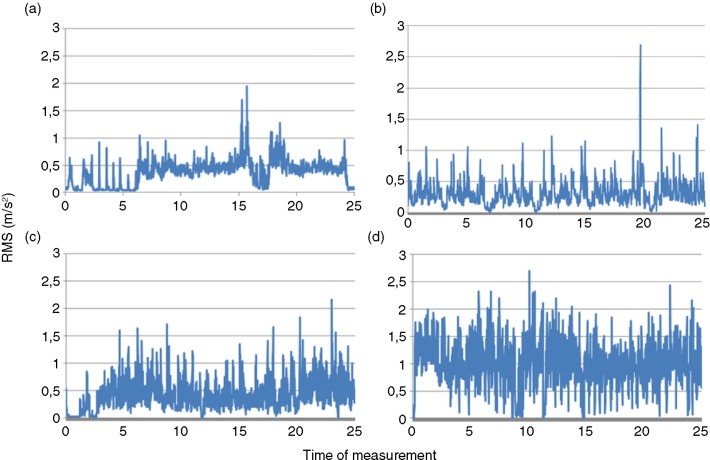
Graph showing the RMS values per second over a 25-min measurement time for 4 common mining vehicles in the Z-direction (upward–downward); (a) haul truck, (b) excavator, (c) wheel loader and (d) track dozer.

Exposure to vibration varies substantially over a work cycle. Figure 1a–d provides graphic representations of RMS values of the measurement time for 4 common mining vehicles to demonstrate the great variability in measured vibration magnitudes. Figure 1a shows the RMS values for a haul truck during the whole work cycle from waiting for the load, loading, driving loaded, dumping and driving unloaded. Figure 1b shows the RMS values for a large excavator while loading 6 haul trucks. Figure 1c shows the RMS values for a wheel loader, and Figure 1d shows the corresponding values for a track dozer. The figures show that different subtasks produce significant different vibration magnitudes and that the RMS value varies from almost 0 up to 2.5 m/s^2^.

**Fig. 2:**
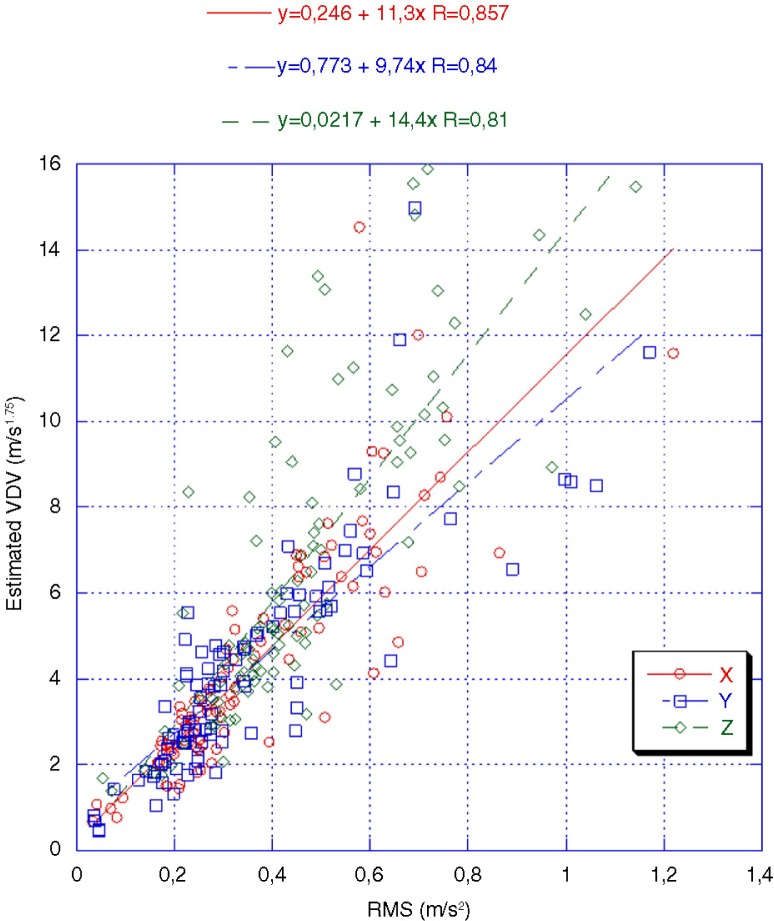
Relationship between measured mean RMS values and estimated mean VDVs for the mean measuring time in the 3 vibration directions separately. The linear regression equations for each direction are also given.

Figure 2 illustrates the relationship between mean measured RMS values and corresponding VDVs calculated for the mean measuring time for each group of vehicle, respectively. The figure also shows the linear regression equations between RMS value and VDV for each direction separately.

There was a close relationship between measured RMS values and VDVs, with a correlation between 0.8 and 0.9 for the different vibration directions. The highest correlation was found in the X-direction, and the lowest in the Z-direction. The linear regression equations show that the increases in VDV values are greatest in the Z-direction and lowest in the Y-direction in relation to the corresponding RMS values.

Of the 453 miners who answered the questionnaire, 283 miners indicated that they were exposed to WBV during their working day. The mean daily exposure time to WBV was 5.1 h (SD 2.8 h; min.–max. 0.1–12.0 h).

In Table II, the reported daily exposure duration for different types of mining vehicles has been compiled. Moreover, the calculated weighted RMS acceleration and VDV are also given for the mining vehicles over an 8-h period.

**Table II:** The reported daily equivalent exposure time for whole-body vibration among the most commonly exposed occupations (questionnaires n=283; the standard deviation is given in parenthesis)

Group of vehicle	Number of answers	Exposure time (hours)	A(8) (m/s^2^)	8-h VDV (m/s^1.75^)
Haul trucks	87	6.2	0.38	8.0
		(1.8)		
Drilling rigs	31	6.3	0.20	8.6
		(2.2)		
Wheel loaders	69	4.6	0.66	17.3
		(2.4)		
Excavators	41	4.8	0.40	11.2
		(2.4)		
Dozers	25	6.6	1.04	14.8
(wheel. track)		(1.3)		
Graders	22	6.7	0.36	9.8
		(2.3)		
Transport cars	170	1.9	0.35	7.3
		(1.7)		

The calculated A(8) as well as the 8-h calculated VDV for each group of vehicles is provided.

The total numbers of answers (n=445) indicated that around one-third of the workers are exposed to WBV from more than 1 vehicle and transport cars in particular are used in a high frequency. The results show that the mean daily exposure time for the different vehicles varies between 1.9 and 6.7 h. The calculated mean A(8) could be found in an interval between 0.2 and 1.0 m/s^2^, and the corresponding 8-h VDV between 7 and 17 m/s^1.75^.

## Discussion

Increased mechanization in mining has resulted in the fact that the proportion of workers exposed to WBV has increased (10), and in the literature, there were numerous studies exploring WBV exposure levels experienced during the mining operation, especially using haul trucks. This study shows that operators of mining vehicles are exposed to potentially harmful levels of WBV, which is in line with earlier findings (2–7). The measured RMS values and VDVs are also consistent with earlier measurements, which indicate that the exposure to WBV in Barents mines do not differ from the exposure that could be found in other mines. However, measurements conducted on haul trucks in Russian open-cast mines in the Barents region (17) showed a mean A(8)-value of 1.0 m/s^2^ and a mean 8-h VDV of 10.4 m/s^1.75^. These values are a little higher than we obtained in our study, but the Russian haul truck drivers were exposed to markedly long durations (12 h), which could explain some of the difference.

The vehicles these measurements were obtained from were randomly sampled from the stock of machines at the different mines, both new and old models. However, the results may not be representative of the exposures experienced by all drivers at the mine sites. Conversely, however, there is no reason to believe that the situations measured were atypical, nor that the mining vehicles were unusual for the industry. It is known that the WBV magnitudes are dependent on the phase of the work cycle; roadway conditions and grade; vehicle speed; vehicle type and size; vehicle suspension and maintenance; tyre design and maintenance; seating design, maintenance and adjustment; and driver behaviour (18). The results obtained in this study show the same pattern and also highlight the need for conducting measurements including all work phases for establishing the daily exposure.

For haul trucks, the measured WBV levels were on average below the action value for A(8) and 8-h VDV, but it is important to notice the wide range of exposure levels and exposure durations. Several earlier studies on WBV on haul trucks in open-cast mines found moderate-to-high levels of WBV exposure (3,5,7,17). Therefore, it is of fundamental importance to reduce WBV levels for operators of these vehicles through regular maintenance of vehicles, levelling of roads and training the operators to use the vehicle in such a way as to reduce the WBV exposure.

Earlier studies on earth scrapers and heavy earthmoving machines in mines found high levels of WBV exposure (4,6). In the current study, drivers of wheel loaders and dozers are exposed to WBV that also exceeds the action limit for A(8) and 8-h VDV, according to the European vibration directive (12), which implies the need for active measures.

What is also noticeable is the high vibration load caused by the use of various transport vehicles (minibuses, vans). These transport vehicles are used, among other things, for the transportation of workers to the various mining vehicles. Normally, the use of these vehicles is limited, but they appear to provide a significant contribution to daily exposure for all workers.

The comparison between mean measured RMS and corresponding VDVs indicates quite a close relationship. This is also in agreement with Wolfgang et al. (7) who also found that the linear relation of the correlation between RMS values and VDVs in the Z-direction was around 0.81 (calculated from their data). The comparison also shows that, particularly, exposure in the vertical up–down direction creates irregular exposure (impacts) that affects the VDVs. Moreover, the calculated daily equivalent exposure, A(8), indicates that drivers of 2 of the vehicle groups are exposed to WBV above the “action value” according to the European vibration directive. When compared with VDVs in the directive, it was found that 4 of the vehicle groups are above the action value. These results show the need to evaluate the vibration exposure in both ways in order to provide a proper risk assessment. It should also be noted that some of the individual measurements for driving dozers exceed the European vibration directive's exposure limit values.

## Conclusions

The measurements conducted of WBV among operators of mining vehicles/equipment at the 3 mines clearly show that exposure to vibration may be a serious health and safety problem that ought not to be ignored. The mines should, therefore, as soon as possible take more active steps to reduce exposure in accordance with the European vibration directive. To evaluate the risk of vibration exposure, the results show that it is necessary to use both the RMS and VDV methods for estimating the risk. The investigation has also pointed out work groups that are exposed to vibrations that are close to or exceed the exposure limit values, for which the employer should take immediate action to reduce exposure below this value. One general piece of advice is to offer all workers exposed to vibration in the mines appropriate regular health monitoring.

## References

[1] Burström L, Nilsson T, Wahlström J (2014). Whole-body vibration and the risk of low back pain and sciatica: a systematic review and meta-analysis. Int Arch Occup Environ Health.

[2] Howard B, Sesek R, Bloswick D (2009). Typical whole body vibration exposure magnitudes encountered in the open pit mining industry. Work.

[3] Kumar S (2004). Vibration in operating heavy haul trucks in overburden mining. Appl Ergon.

[4] Salmoni A, Cann A, Gillin K (2010). Exposure to whole-body vibration and seat transmissibility in a large sample of earth scrapers. Work.

[5] Smets MP, Eger TR, Grenier SG (2010). Whole-body vibration experienced by haulage truck operators in surface mining operations: a comparison of various analysis methods utilized in the prediction of health risks. Appl Ergon.

[6] Vanerkar AP, Kulkarni NP, Zade PD, Kamavisdar AS (2008). Whole body vibration exposure in heavy earth moving machinery operators of metalliferrous mines. Environ Monit Assess.

[7] Wolfgang R, Burgess-Limerick R (2014). Whole-body vibration exposure of haul truck drivers at a surface coal mine. Appl Ergon.

[8] Mandal BB, Srivastavab KA (2010). Musculoskeletal disorders in dumper operators exposed to whole body vibration at Indian mines. Int J Min Reclamat Environ.

[9] Skandfer M, Talykova L, Brenn T, Nilsson T, Vaktskjold A (2014). Low back pain among mineworkers in relation to driving, cold environment and ergonomics. Ergonomics.

[10] McPhee B (2004). Ergonomics in mining. Occ Med.

[11] ISO2631-1 (1997). Evaluation of human exposure to whole-body vibration. Part 1-General Requirements.

[12] Directive 2002/44/EC (2002). Directive 2002/44/EC of the European parliament and of the Council of 25 June 2002 on the minimum health and safety requirements regarding the exposure of workers to the risks rising from physical agents (vibration) (sixteenth individual directive within the meaning of Article 16(1) of Directive 89/391/EEC). Off J Eur Communities.

[13] EN 14253 (2003). Mechanical vibration – measurement and calculation of occupational exposure to whole-body vibration with reference to health – practical guidance.

[14] MineHealth (2012). Sustainability of miners’ well-being, health and work ability in the Barents region – a common challenge. Kolarctic ENPI CBC Contract no. 02/2011/043/KO303.

[15] Burström L, Sjåland C, Ivkova N, Björ B, Johnsen M, Hyvärinen V (2012). Measurements of hand-transmitted and whole body vibration. Working Document No. WP2- D1:1, MineHealth project “Sustainability of miners’ well-being, health and work ability in the Barents region – a common challenge,” KOLARCTIC ENPI CBC Grant Contract no. 02/2011/043/KO303.

[16] MineHealth Consortium (2012). Questionnaire – all questions. Working Document No. WP2_D31, MineHealth project “Sustainability of miners’ well-being, health and work ability in the Barents region – a common challenge,” KOLARCTIC ENPI CBC Grant Contract no. 02/2011/043/KO303.

[17] Øvrum A, Skandfer M, Siurin S, Talykova L, Nikanov A (2012). European and Russian methods for exposure assessment applied on whole body vibration values in short Haul dump trucks. Ecologiia Tsjeloveka.

[18] Eger T, Salmoni A, Cann A, Jack R (2006). Whole-body vibration exposure experienced by mining equipment operators. Occ Ergon.

